# Unlocking potential: a qualitative exploration guiding the implementation and evaluation of professional role substitution models in healthcare

**DOI:** 10.1186/s43058-024-00611-x

**Published:** 2024-07-12

**Authors:** Rumbidzai N. Mutsekwa, Katrina L. Campbell, Russell Canavan, Rebecca L. Angus, Liza-Jane McBride, Joshua M. Byrnes

**Affiliations:** 1 Gold Coast Hospital and Health Service, Nutrition and Food Services, 1 Hospital Boulevard, Southport, Queensland 4215 Australia; 2 Gold Coast Hospital and Health Service, Allied Health Research Team, 1 Hospital Boulevard, Southport, Queensland 4215 Australia; 3 Centre for Applied Health Economics, School of Medicine, Griffith University, Sir Samuel Griffith Centre, 1 Parklands Drive, Nathan, Queensland 4111 Australia; 4 Menzies Health Institute Queensland, Griffith University, Gold Coast Campus, 1 Parklands Drive, Southport, Queensland 4215 Australia; 5 Healthcare Excellence and Innovation, Metro North Hospital and Health Service, 153 Campbell Street, Bowen Hills, Queensland 4029 Australia; 6 Gastroenterology Department, Gold Coast Hospital and Health Service, 1 Hospital Boulevard, Southport, Queensland 4215 Australia; 7 School of Health Sciences and Social Work, Griffith University, Gold Coast Campus, 1 Parklands Drive, Southport, Queensland 4215 Australia; 8 Department of Health, Clinical Excellence, 15 Butterfield Street, Herston, Queensland 4006 Australia

**Keywords:** Professional role substitution, Scope of practice, Implementation, Evaluation, Measuring performance

## Abstract

**Background:**

As role substitution models gain prominence in healthcare, understanding the factors shaping their effectiveness is paramount. This study aimed to investigate factors that impact the implementation and performance evaluation of professional role substitution models in healthcare, with a focus on understanding the variables that determine their success or failure in adoption, execution, continuity, and outcomes.

**Methods:**

The exploratory qualitative study used semi-structured interviews with key opinion leaders, decision makers, facilitators, recipients, and frontline implementers, who had influence and involvement in the implementation of professional role substitution models. Data analysis was guided by the Consolidated Framework for Implementation Research (CFIR).

**Results:**

Between November 2022 and April 2023, 39 stakeholders were interviewed. Factors influencing implementation and evaluation of allied health professional role substitution models of care aligned with the five core CFIR domains (innovation, outer setting, inner setting, individuals, implementation process) and outcome domain incorporating implementation and innovation outcomes. The six themes identified within these CFIR domains were, respectively; i) Examining the dynamics of innovation catalysts, evidence, advantages, and disadvantages; ii) Navigating the complex landscape of external factors that influence implementation and evaluation; iii) Impact of internal structural, political, and cultural contexts; iv) The roles and contributions of individuals in the process; v) Essential phases and strategies for effective implementation; and vi) The assessment of outcomes derived from allied health professional role substitution models.

**Conclusions:**

The study highlights the complex interplay of contextual and individual factors that influence the implementation and performance evaluation of professional role substitution models. It emphasises the need for collaboration among diverse stakeholders to navigate the challenges and leverage the opportunities presented by expanded healthcare roles. Understanding these multifaceted factors can contribute to the development of an empowered workforce and a healthcare system that is more efficient, effective, safe, and sustainable, ultimately benefiting patients.

**Supplementary Information:**

The online version contains supplementary material available at 10.1186/s43058-024-00611-x.

Contributions to literature • There is limited understanding of the complex interplay of contextual and individual factors that influence implementation and performance evaluation of professional role substitution models of care.

• This study provides comprehensive guidance on successful implementation and evaluation of new models of care which influences efficient use of resources in healthcare.

• This study contributes to recognised gaps in literature, seeking to demonstrate value proposition of professional role substitution models of care. This study has identified outcome measures that can determine the successful implementation and impact of these models of care

## Background

The healthcare sector plays a crucial role in ensuring the well-being of individuals and society, but it is facing challenges due to a growing and ageing population. The demand for high-quality healthcare has increased significantly, while the shortage of healthcare workers has become a pressing concern [1, 2]. Workforce reforms are now being prioritised in healthcare to shape the future of healthcare delivery. These reforms include initiatives to increase the number of healthcare workers, enhance the quality and duration of healthcare education and training, and diversify the healthcare workforce.

One key strategy to address healthcare challenges is the expanded scope of practice for non-medical healthcare professionals [3]. This expansion entails a discrete knowledge and skill base beyond the recognised scope of practice within a specific jurisdiction's regulatory framework [4]. It empowers healthcare practitioners such as nurse practitioners, allied health professionals, and physician assistants to practice to the full extent of their training and education, or to extend their scope of practice beyond traditional boundaries [5–7]. Consequently, they can perform a broader range of tasks, including those previously reserved for medical doctors.

Professional role substitution models have improved patients' access to healthcare services [8–10]. Moreover, there is a growing body of evidence suggesting that these alternative healthcare delivery models can provide safe and effective care that patients find acceptable. Nurse practitioners and advanced nurses in the US, Canada, the UK, and Australia expand primary care roles, including diagnosis, prescribing, patient education, managing long-term conditions, and minor surgeries [9, 11–13]. Physician assistants (PAs) in countries like the US, Canada, and the Netherlands work closely with physicians, conducting assessments, diagnosing, treating common illnesses, and providing patient education. PAs improve healthcare access, especially in underserved and rural areas with physician shortages [14–16].

In developing countries with limited healthcare resources, professional role substitution models are vital for addressing shortages of skilled healthcare providers and improving access to essential services. For instance, in sub-Saharan Africa, task shifting from physicians to nurses and community health workers addresses the scarcity of skilled providers [17, 18]. Community health workers, trained to deliver basic healthcare services and education, play crucial roles in preventive and promotive interventions, particularly in rural and underserved areas [18]. Nurse-led clinics have also proven successful in delivering comprehensive primary care services, such as antenatal care and family planning, alleviating pressure on strained healthcare systems [19–21].

Allied health professionals, encompassing disciplines such as speech pathology, pharmacy, dietetics, physiotherapy, occupational therapy, radiography, sonography, psychology, and social work, are increasingly vital in diverse healthcare settings. Supported by mounting evidence of their effectiveness, their role continues to expand [10, 22]. Despite substantial growth, particularly notable in Australia where they rank as the second-largest healthcare group, [23] the implementation of professional role substitution within allied health is relatively new compared to fields like nursing and physician assistants [24].

The successful implementation of all professional role substitution models including allied health is complex and contingent on various factors which are not currently well understood or defined [10, 22, 25]. To ensure success, it is essential to consider the impact on patients, healthcare professionals, and the healthcare system [25]. This must be approached from a multi-stakeholder perspective, involving experts in the field, key opinion leaders, healthcare leaders, decision makers, policy makers, recipients, and frontline implementers.

Research into the expanded scope of practice within allied health disciplines, including implementation and performance evaluation, is crucial [6, 23, 25, 26]. Previous studies have highlighted patients' perceptions and experiences of healthcare quality in role substitution models [27, 28]. While clinicians express support for performance evaluation, there's a gap between support and effective implementation [29]. There's also a lack of agreed-upon approaches for measuring performance [25, 29]. Collaborative efforts involving multiple stakeholders are essential for understanding robust evaluation methods and optimising alternative models of care for healthcare transformation and sustainability [25].

To address this gap in knowledge and practice, this study aimed to describe the individual and contextual factors that influence the implementation and performance evaluation of allied health professional role substitution models from a multi-stakeholder perspective. Furthermore, the study aimed to identify outcome measures that can demonstrate the successful implementation and impact of these models of care.

## Methods

### Study approach and design

An exploratory qualitative approach was used to explore expectations, perceptions, and experiences of stakeholders involved in the implementation and performance evaluation of professional role substitution models of care. Semi-structured interviews were chosen as the primary method of data collection to allow for flexible exploration of specific topics and issues, maximising the richness of the data [30]. The study adhered to the Consolidated Criteria for Reporting Qualitative Research (COREQ) guidelines [31]. Please see Additional file 1

### Study setting

This study was conducted within the public healthcare system of the State of Queensland, Australia. This comprises 16 hospital and health services and approximately 35,000 allied health professionals [32]. Queensland initiated an allied health strategy in 2014 to expand professionals' scope of practice, resulting in the establishment of 133 distinct models of care by 2019 [24, 33, 34]. Examining this system offers valuable insights into implementing and evaluating professional role substitution models, providing practical understanding within a specific healthcare context.

### Study participants and recruitment

A purposeful sampling strategy was employed to recruit key stakeholders at various levels of the healthcare system who were involved in some way in the implementation and performance evaluation of allied health professional role substitution models of care. Participants included experts in the field, key opinion leaders, decision makers, recipients, and frontline implementers, implementation facilitators and support teams. A sampling matrix was used to consider factors such as location, affiliation, organisational role, tenure, and profession ensuring diversity and representation across the different dimension of the healthcare system. While a specific target number of participants was not predetermined, our aim was to achieve saturation in the sample, ensuring comprehensive coverage of perspectives and experiences relevant to our research objectives. Email invitations were sent to potential participants/participant groups, along with study information and consent forms. Those who agreed to participate contacted the principal investigator to arrange a suitable interview time.

### Positionality of researchers

The research team comprised individuals with diverse backgrounds and roles, including experts in professional role substitution, health services research, economics, qualitative study methodology, and healthcare management.

### Ethics

This study was performed in line with the principles of the Declaration of Helsinki with approval granted by Gold Coast Hospital and Health Service (HREC/2020/QGC/62104) and Griffith University (GU Ref No: 2020/876). All participants provided written informed consent.

### Data collection

An interview guide was developed by the research team to ensure coverage of the study aims and objectives (Additional file 2). The guide was pilot tested with three eligible participants, resulting in minor wording adjustments for clarity. Interviews were conducted either face-to-face or via video conferencing with only interviewer and participant present. The semi-structured interviews were designed to elicit open-ended responses from participants, with the interviewer using prompts and probing techniques as needed. Data collection continued until data saturation was reached, indicating that no new themes were emerging [35]. All interviews were audio-recorded, transcribed, and supplemented with field notes for additional context and consistency. Each participant was allocated an anonymous identifier, comprising their participant number along with a descriptor of their role or professional background. (e.g., P34, Workforce and Education). Participants were offered the opportunity to check their transcript.

### Data analysis and interpretation

Descriptive statistics were used to analyse demographic data, such as participants' time in their current role, age, gender, and education level. Exploration of contextual influences on implementation and performance evaluation was guided by the Consolidated Framework for Implementation Research (CFIR) [36]. The CFIR is a comprehensive framework that focuses on understanding and improving the implementation and evaluation of health innovations. Its adaptability enables integration into various contexts, fostering analysis and facilitating cross-study comparisons. This versatility supports a systematic approach to evaluating implementation processes and outcomes, thereby enriching our understanding of innovation dynamics across diverse settings [36].

It consists of six domains: 1. Innovation domain (the model of care being implemented), 2. Outer setting (the healthcare system in which the inner setting exists) 3. Inner setting (the site in which the model of care is implemented e.g., hospital) 4. Individuals (the roles and characteristics of individuals involved in the implementation process), 5. Implementation process (the activities and strategies used to implement the model of care), 6. Implementation outcomes (perceptions and measures of implementation success or failure), and Innovation outcomes (outcomes that capture success or failure of model of care) [36–38].

A reflexive thematic approach was taken for qualitative analysis [39]. The analysis began deductively with codes derived from the CFIR, followed by inductive coding to identify additional categories. These codes were assigned using CFIR definitions, inclusion/exclusion criteria, and appropriate quote examples. NVivo V10 software (QSR International Ltd.) was used to facilitate data management.

Investigator triangulation was employed, with the principal researcher (R.N.M) coding all interviews and 20% of the interviews coded by a second researcher (R.L.A) to enhance reliability and provide different perspectives [40]. All authors participated in summarising codes prioritised for analysis and interpreting the results. A matrix was created to compare the ratings of each CFIR construct, focusing on any differences among stakeholders. Data extracts were selected to illustrate themes and subthemes, incorporating multiple perspectives for interpretation.

## Results

### Study population

A total of 39 stakeholders from various hospital and health services across Queensland were interviewed. The stakeholders represented a broad spectrum of positions and roles within the healthcare system, categorised into eight groups: allied health clinicians, medical practitioners/general practitioners, nursing staff, allied health leadership, hospital and health services/statewide leadership, recipients, implementation support personnel, workforce and education. Table 1 provides demographic details of the participants.

**Table 1:** Participant characteristics

**Demographic**	**Total (*n*=39)**
**Age, years n (%)**	
26-35	5(12.8)
36-45	16(41.0)
46-55	12(30.8)
56-65	4(10.2)
>65	2(5.1)
**Gender n (%)**	
Male	12(30.8)
Female	27(69.2)
**Highest Education Level n (%)**	
Bachelor’s degree	9(23.1)
Graduate certificate	7(17.9)
Graduate diploma	3(7.7)
Master’s degree	15(38.5)
Doctoral degree	5(12.8)
**Professional Roles n (%)**	
*** Allied Health Clinicians***	
Dietitian/nutrition	2(5.1)
Occupational therapy	2(5.1)
Pharmacy	1(2.6)
Physiotherapy	1(2.6)
Podiatry	1(2.6)
Radiography	1(2.6)
Speech pathology	2(5.1)
Clinical measurements	1(2.6)
*** Medical Practitioners /Nursing***	
Specialist clinical/medical directors	3(7.7)
General Practitioners /Liaison	2(5.1)
Nursing practitioner	1(2.6)
*** Allied Health Leadership***	
Allied health executive directors / professional directors	4(10.3)
Allied health middle managers	2(5.1)
*** Hospital and Health Services/ State-wide Leadership***	
Board, executive and clinical senate representatives	3(7.7)
Healthcare purchasing and policy development	2(5.1)
*** Recipients***	
Consumers/Consumer Representatives	2(5.1)
*** Workforce and Education***	
University representatives	2(5.1)
Professional /workforce and union body representatives	3(7.7)
*** Implementation support***	
Health strategy, digital and transformation	2(5.1)
Clinical research fellows and support	2(5.1)

Participants had been in their roles on average 11 years, (range 1-27 years). Interviews had an average duration of 32 minutes (range 15-59 minutes). Five interviews were conducted face to face with the remainder (*n*=34) conducted through video conferencing.

### Findings

**Fig. 1:**
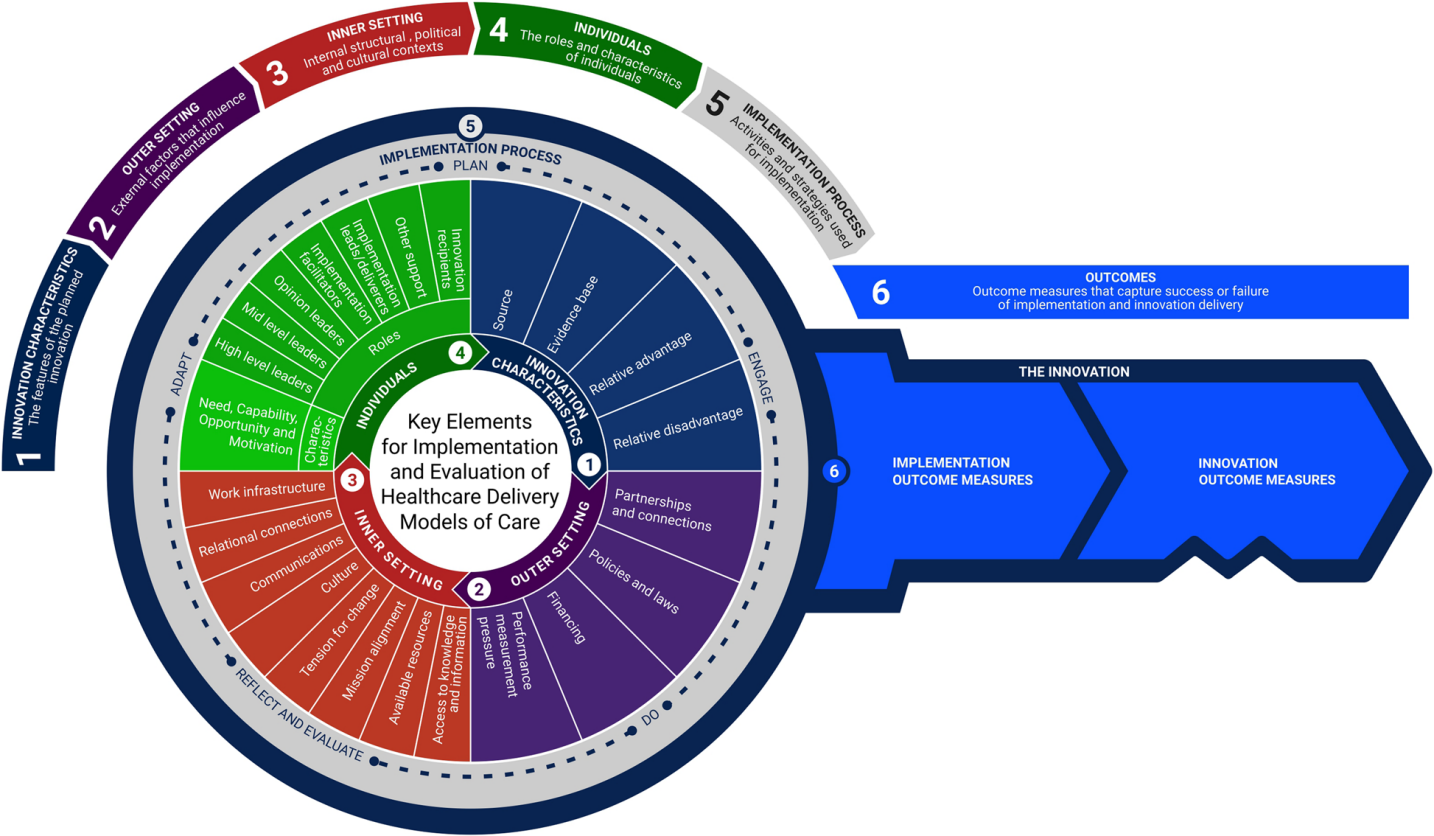
Key implementation and evaluation constructs for professional role substitution models of care

Six themes were identified which aligned with the five CFIR domains and the outcomes domain. Twenty-seven underlying constructs and subconstructs of the CFIR were identified as factors influencing implementation of professional role substitution in our analysis. Ten constructs were identified in the implementation and innovation outcome categories. Main domains and constructs are illustrated in Fig. 1.

## Innovation domain

### Examining the dynamics of innovation catalysts, evidence, advantages, and disadvantages in allied health professional role substitution models of care

The following section delineates the three primary constructs aligning with CFIR domains and an additional domain, namely "relative disadvantage." These constructs were identified from the perspectives of participants regarding professional role substitution models of care as an innovative approach.

#### Innovation source

Participants recognised healthcare system strain due to workforce shortages, rising costs, and increased needs with policies now prioritising workforce reform as a key healthcare strategy. The 2006 Australian Productivity Commission review, focusing on optimising scope, competencies, and job redesign was frequently cited by interviewees as the catalyst for change. The Ministerial Taskforce on Health Practitioner Expanded Scope of Practice (Queensland), alongside similar taskforces nationwide, played a vital role in implementing allied-health professional role substitution models of care. *“There were a broad range of stakeholders involved in the task force across Queensland Health and external to Queensland in 2014. There was a number of recommendations in the report with overall endorsement from the Minister.”* (P34, Workforce and Education)

Furthermore, the Allied Health Professions' Office supported these efforts by funding care models, addressing legislative barriers, developing training, supporting research, monitoring progress, and sharing achievements. A participant explained, *‘The office was charged with implementing the recommendations and to test these models of care. Particularly things like requesting and interpreting forms for diagnostic imaging and requesting pathology.” (*P34, Workforce and Education)

#### Evidence base

Participants expressed varying perspectives on the evidence base for professional role substitution models of care. Some noted a reliance on grey literature or information from pilots, highlighting the limited evidence supporting certain models. Conversely, others believed the evidence base was robust and questioned the need for further piloting. *“It should be business as usual and that’s something we’ve tried to promote where we’ve got evidence from other jurisdictions and internationally. There should then be efforts to implement and try and replicate those results and take it to scale.”* (P34, Workforce and Education).

Established services in other countries and professions influenced the implementation in Australia. Clinician leads or facilitators with prior experience in allied health models were identified as key enablers of this process. One participant shared their experience stating, *“I was involved with that over there in the UK and so I came with that mentality to Queensland. When I worked as a fellow, I was surprised that there wasn’t that model, and I advocated for it and was told we don’t do that here. So, we ended up running extra clinics as fellows to see the long-wait patients when I knew that back in the UK it would have been [allied health discipline*].” (P19, Medical Specialist)

#### Relative advantage

Participants, healthcare professional and patients alike identified several advantages of allied health professional role substitution models. These models improved access to care, particularly benefiting underserved areas, and boosted efficiency by *“streamlining decision-making and minimising duplication”* (P19, Medical specialist). A patient shared their positive experience, stating, *“If anything, I thought I was really special. I got pushed ahead really. I didn’t have to wait so long, and I wasn’t made to feel silly for my symptoms and they were investigated. The whole experience was positive.” (*P39, Consumer/Recipient)

While considered cost-effective by those interviewed due to reduced reliance on specialists in resource-limited settings, many highlighted the need for further cost-effectiveness data. One participant mentioned,

*“You can get comparable or sometimes a better service at a lower cost using alternate models of care.” *(P17, Medical Specialist)

Participants indicated that these models enabled allied health professionals to provide comprehensive, patient-centred care, enhancing overall healthcare experiences and patient satisfaction. Another viewpoint shared was, “*It's about getting patients to clinicians with holistic skill sets rather than just the medical model. Traditionally, patients wait a long time to see a medical professional, only to be referred back to the same clinician “, (*P15, Implementation Support). Additionally, clinicians working in these roles noted*, “So, they’re kind of getting that one stop assessment, where the speech pathologist looks at the functional component, as well as pathology or organic disease”* (P13, Allied Health Clinician). Moreover, they promoted professional growth, job satisfaction, and workforce retention through expanded roles and skill development opportunities, fostering collaboration among healthcare professionals from various disciplines for improved patient outcomes. One individual expressed *“That responsibility and that extra challenge for me is where I get the buzz. (P*12, Allied Health Clinician*)*

#### Relative disadvantage

In addition to the benefits of professional role substitution in healthcare, participants emphasised other key factors. Patient safety and care quality surfaced as paramount concerns. A participant with workforce and education background stated, *“There was a lot of the discussion and particularly the negative media coverage around the model of care. I was quite driven to answer the questions, or the concerns raised by the health professionals around safety*.” (P29, Workforce and Education)

The imperative of ensuring skill, competence, and appropriate clinical governance was strongly emphasised. In some settings, participants flagged the potential for resistance and conflicts with traditional providers and organisations, driven by apprehensions about expertise encroachment, de-skilling, and role ambiguity. A Medical Specialist (P19) highlighted this, *“The risk is that if you promote therapists from being treating therapists to being screening and treating therapists, you’re on the risk of deskilling your (medical) workers.”*

Building public and patient trust, especially in unfamiliar models, highlighted the importance of transparent communication and educational efforts, as noted by both consumers and healthcare professionals. A patient shared, *“I really didn’t know what to expect because I hadn’t been to a clinic like that before and I didn’t know what they were going to do”.* (P39, Consumer/recipient). A healthcare professional suggested, “*Another barrier is patient perception, especially if they are expecting to see a doctor”* but went on to add*, “In my experience this has often not been the case with patients often reassured once they have had a thorough assessment*” (P13, Allied Health Clinician) Initial challenges in interaction with General Practitioners (GPs), were also highlighted with one participant noting, “*See the problems at the beginning where the GPs would ring up and say, I wanted a specialist opinion, and I got a physiotherapist. But once they were educated, those complaints dropped off especially when the patient satisfaction scores were high”* (P19, Medical Specialist).

The implementation of these models often demands additional investments in training and supervision, with a consideration of their economic and logistical impact on the healthcare system required. Lastly, *“striking a delicate balance between expanded scopes and core responsibilities”* (P 25, Allied Health Leadership) is essential. Another participant noted, “*It is also worth considering the amount of time it takes for this training and to set up these roles. It is also important to consider the cost. Once you have a model of care set up well, what’s my sustainability plan for this model in relation to, succession planning, leave management, etcetera*? “(P15, Implementation Support)

## Outer setting

### Navigating the complex landscape of external factors that influence implementation and evaluation of allied health professional role substitution models of care

#### Partnerships and connections

Collaborative care teams and strong referral networks emerged as crucial elements for successful role substitution practice. Participants emphasised the importance of interdisciplinary collaboration, where professionals from various disciplines worked together to provide holistic patient care. Furthermore, partnerships with specialists, hospitals, community resources, and primary care facilities were highlighted as essential for ensuring seamless transitions and continuity of care. This was articulated by one GP, (P7) *“I think for me and my style of medicine, it’s helpful. I really enjoyed that sort of team, that real MDT and holistic approach to patient care.”*

#### Policies and laws

Implementing professional role substitution and scope extension may require legal and regulatory adjustments, including redefining boundaries and establishing standards which participants noted as a challenge. Variations across jurisdictions, were highlighted emphasising the need for a national approach to align state and federal policies. An occupational therapist identified legislative barriers stating, *“Legislation prevents us from ordering imaging, but we all have local agreements with our departments that enable us to order basic radiology. But we want to be able to order that radiology in our general role as well and potentially expand that into other forms of the imaging down the road. This role has expanded even further in the UK to some of those therapists prescribing and referring people for MRIs and CT scans.”* (P11, Allied Health Clinician)

Both allied health clinicians and medical doctors expressed concerns about legal accountability in the event of adverse events or complications in professional role substitution models. Stakeholders, including allied health clinicians, medical doctors, and healthcare leaders, emphasised the importance of assurance of indemnity through health services. *“We’re protected by public indemnity in this system. And ultimately the directors are responsible for all the patients, even the ones we don’t directly treat. So that model protected our junior doctors and subsequently protects the therapists as well*” (P19, Medical Specialist). Participants also acknowledged the need for regular training and re-assessment of knowledge and skills for medical professionals but were uncertain about the lack of similar scrutiny and regulation mechanisms for allied health clinicians in professional role substitution roles.

#### Financing

Participants had differing perspectives on funding for new models of care. Implementing professional role substitution models of care often relied on short-term funding and grants to pilot services. A participant with an allied health clinician background highlighted complexities in healthcare funding and incentives, pointing out *“General practice won’t make money unless the patient sees the GP. They would need to look at some sort of MBS (Medicare Benefits Schedule) item number so that the practice or hospital and health service can generate money from those expanded roles.”* (P10, Allied Health Clinician)

Suggestions were made to review Medicare and activity-based funding structures to provide support for professional role substitution models ensuring their viability. A participant who has supported implementation of a professional role substitution model noted, *“That’s also based on the fact that with Activity-Based Funding framework, we have to demonstrate that the model can generate enough activity to be viable and valuable.”* (P15, Implementation Support).

Additionally, participants emphasised the importance of funding models that prioritise outcomes rather than specific care delivery mechanisms. A healthcare executive highlighted, “W*e don’t purchase models of care. I would like to think that we purchase outcomes, and we are quite agnostic in how health services go about achieving those outcomes. We’ve wanted to make sure that the funding model is enabled and that it’s not a barrier to people trying alternative ways using new and different models to achieve those outcomes that we’re interested in.”* (P32, Hospital and Health Services/ State-wide Leadership).

#### Performance management pressure

Participants acknowledged the challenge of meeting patient waiting time targets set by federal and state governments. This was an enabling factor, with professional role substitution models of care implemented as strategies to reduce specialist outpatient waitlists and improve access to services, aligning with performance targets. *“There was a wait list issue for the specialty area. There was a big project to see who else could help see patients and try and reduce the waitlists. They highlighted that the [allied health specialist area clinician] might be something that could help with that.”* (P8, Allied Health Clinician).

## Inner setting

### Impact of internal structural, political, and cultural contexts on the implementation and performance evaluation of allied health professional role substitution models in healthcare

#### Work infrastructure

Implementing role substitution models had workforce implications, including assessing skills availability and workload management. Sustainability relied on individual commitment, posing threats to the longevity of these models of care. An allied health leader, (P22) noted, *“Often the first people you get in are personally passionate about it. It’s hard to find those people all the time, but a succession plan is important for sustainability of extended scope roles.“* These sentiments were echoed by a physician who mentioned, *“Workforce and sourcing the right resources and clinicians is something that is a bit of a challenge for the health services moving forward.”* (P17, Medical specialist)

#### Relational connections

Participants identified strong relationships and networks as vital for implementing and sustaining professional role substitution models. Trust between medical doctors and allied health clinicians was essential. As one participant noted: *“Most of the time when these models fall down, it’s because the relationships between the allied health and the multidisciplinary team, including the doctors, have broken down. The doctor’s left or there’s been an issue that they couldn’t resolve and then everything falls to pieces.”* (P23, Allied Health Leadership) Key roles of advocates and clinical leads were emphasised, but overreliance on individuals was a concern. Building resilience in these models across all levels of leadership was an important consideration as are clear governance structures which include supervision and escalation pathways.

#### Communications

Effective communication was necessary for high-quality care, patient safety, and collaborative relationships in both implementing and sustaining professional role substitution models. Iterative modifications and a willingness to learn were recognised as important. Collaboration involved shared decision-making, regular communication, and joint management of complex cases. Specialist doctors provided guidance and medical expertise, while allied health professionals contributed their specialised skills including ability to provide holistic care.* “We still needed to iron out all of the kinks, so each side still needs to continue to learn from each other. So, I would say it probably took a good 12 to 18 months before we felt like we had a system that was working well for both sides and streamlining the process.”* (P11, Allied Health Clinician). Additionally, some participants emphasised transitioning from *“substitution-focused to team-based approaches”* (P27, General Practitioner and Healthcare Executive), promoting interdisciplinary and transdisciplinary care.

#### Culture

For some participants, professional role substitution raised concerns about autonomy with potential for conflicts among healthcare professionals. Cultivating a collaborative culture, renegotiating traditional hierarchies, and addressing professional dynamics were identified as strategies to enable interprofessional collaboration, promoting innovation and excellence in patient care. However, despite the progress made, some participants expressed reservations about barriers that still exist, even in allied health practitioners performing tasks that were within their scope of practice. One executive leader expressed frustration at the slow pace of change stating, “*It’s an imperative at the moment that we actively promote full scope of practice and give more support for our allied health staff to do extended scope of practice qualifications. So, we have a role to ensure that we have a culture that encourages the new models of care, because just to have the old models of care, it’s not simply sustainable, it’s not sustainable, at all.*“ (P36, Hospital and Health Services/ State-wide Leadership)

Another participant, an allied health leader (P22), highlighted the positive impact of professional role substitution on organisational culture and the morale of younger professionals, stating, *“It’s good for our culture and gives some sort of energy to the younger professionals. It also flows through to junior doctors particularly working alongside a consultant that already holds these clinicians and models of care in high standard.”*

#### Mission alignment and tension for change

In many organisations, clinical demand drove professional role substitution adoption, facilitated by change management teams and frameworks. Professional role substitution models aligned with healthcare organisational goals and objectives, promoting innovation, equity, and sustainable use of resources. As articulated by a Medical Specialist (P19)* “We have a limited number of specialists, and training for medical students and junior doctors hasn't significantly increased to meet demand. With advanced technology and reduced working hours, we need to expand services. Having other clinicians who can treat patients without surgery is invaluable.”*

Additionally, participants acknowledged their role in healthcare delivery to underserved communities and advancing health equity in First Nations, rural, and regional areas, “*improving access and preventing, fragmented care*,” (P33, Nursing Health Professional). Furthermore, participants discussed the impact of population growth on surgical waitlists, revealing the pressing need for effective solutions to address increasing demand. An allied health clinician (P11) highlighted the challenges posed by population growth, stating, “*There's been a significant increase in people moving to Queensland now for many years and our surgical wait lists were continuing to grow. So, when I started in this role… the waitlist was almost four years long.”*

Participants stressed the strategic importance of expanded scope in advancing organisational objectives. An Allied health leadership participant (P25), emphasised the multifaceted benefits of expanded scope, highlighting its alignment with strategic goals and the need to reassess care delivery models: *“Expanded scope hits all the strategic goals really. We need to disinvest in some of the low value care because we know that we’re not getting any outcomes. We also need to look at the impact of these models of care.”*

#### Available resources

Funding for professional role substitution models varied, with some implemented without dedicated funding which posed challenges in attracting skilled clinicians. Stakeholders recognised the benefits of co-locating allied health clinicians and medical doctors for interdisciplinary case discussions but sometimes faced challenges due to high demand for limited space. An allied health clinician (P8) highlighted the impact of dedicated funding on the feasibility and efficiency of implementing such models *“They had a certain amount of funding for this project to set it all up. And I think that really made it feasible. So, then we got the right equipment, the right time to set it up. It was a very set process with money attached to it that got it off the ground quicker.”*

#### Access to knowledge and information

Clinicians in extended scope roles actively sought professional development opportunities to expand their skills. Local credentialing and on-the-job training were the norm. A workforce development officer highlighted the rigorous process of credentialing for clinicians in such roles. *“Our credentialling package is fairly intense. It takes months and months and months to become credentialed in a first point of contact clinic like this and needs [Health Service] approval before a clinician can work in a space like this.”* (P29, Workforce and Education). In contrast to nurse practitioner programs offered by universities and specialised training institutions, formal education programs for allied health professionals were scarce. Many participants recommended development of formalised training and credentialing programs to ensure high quality and safe care. *“We’re now in the process of developing our own course here in Australia in collaboration with the university in New South Wales so that we can provide that level of education that we need in these advanced scope roles*” (P11, Allied Health Clinician).

## Individuals domain

### The roles and contributions of individuals in the implementation of allied health professional role substitution models of care

The implementation of allied health professional role substitution models of care heavily relies on the engagement of various individuals who play pivotal roles in the process. Through our interviews, participants identified nine key roles integral to the implementation and evaluation of these alternative healthcare delivery models. These roles, aligned with those in the individuals’ domain of the CFIR, encompassed high-level leaders, mid-level leaders, opinion leaders, implementation facilitators, implementation leads, implementation team members, other implementation support, innovation deliverers, and innovation recipients. Our analysis revealed representation across these roles within our study population, demonstrating the diverse range of contributions.

Participants described the characteristics of these individuals, which we analysed based on the Capability, Opportunity, Motivation-Behaviour (COM-B) theoretical behaviour change model integrated into the CFIR framework. This system evaluates individuals' influence on the implementation process across four constructs: Need, Capability, Opportunity, and Motivation. These constructs assess individuals' deficits addressed by the models of care, their interpersonal competence, availability and power, and commitment and motivation in fulfilling their roles respectively.

Participants emphasised the critical role of medical and executive buy-in for the success of these models. Without their support and commitment, implementation efforts often faced significant hurdles. As one participant stated, "*Medical and executive buy-in, if they are not supportive, it doesn’t happen*" (P34, Workforce and Education). Furthermore, participants highlighted the importance of strong endorsement from medical professionals and the need for active engagement from allied health clinicians and managers to ensure the sustainability of these models. As articulated by another participant, "*Allied health clinicians and even up into the level of our managers, there's certain spheres of influence that we have, but to make something like this come together and to be able to make it sustainable, you really need strong medical endorsement and that real commitment to push it*" (P12, Allied Health Clinician).

Moreover, participants identified the Allied Health Office as having a crucial role in facilitating implementation. However, they also expressed the need for greater visibility and recognition of successful implementation efforts. As one participant suggested, "*The Allied Health Office has a role to play in that. I think we should certainly see more things up in lights, you know, presentations, success stories et cetera and opportunities for these models to be shared and celebrated more widely across the state*" (P15, Implementation Support).

A matrix analysis (Table 2) provides detailed insights into the roles and characteristics of individuals within different groups/roles. This elucidates their contributions to the successful implementation of professional role substitution models of care, as perceived by the study participants.

**Table 2:** Individuals’ roles and characteristics required for implementation and sustainability of professional role substitution models of care

**Roles:**	**Characteristics:**			
	1. Need Experience the following constraints that may be addressed by role substitution:	2. Capability Interpersonal competence and knowledge to:	3. Opportunity Required availability and power to:	4. Motivation Commitment and motivation to:
A. High-level Leaders *Individuals with a high level of authority, including key decision-makers, executive leaders, or directors*	Growing healthcare demand *•* Increasing complexity in healthcare needs *•* Higher consumer/patient expectations *•* Growing healthcare costs *•* Delivery of quality healthcare within budgetary constraints	*•* Execute healthcare system priorities and desired outcomes *•* Identify system enablers to support delivery of strategic goals	*•* Make decisions for resource and funding distribution to endorse, adopt, implement, and sustain models. *•* Execute service level agreements for implementation and reporting on professional role substitution models of care	*•* Contribute to strategic objectives *•* Meet strategic key performance indicators *•* Meet consumer needs with delivery of quality healthcare *•* Develop the workforce *•* Enable positive workforce culture *•* Build a sustainable healthcare system
B. Mid-level Leaders *Individuals with a moderate level of authority, including leaders supervised by a high-level leader who supervises others.*	*•* Supporting high-level leaders to deliver on strategic objectives and goals.	*•* Recruit implementation leads and deliverers. *•* Negotiate resources and distribution *•* Mediate high-level strategy and day to day activities *•* Disseminate strategic information to their teams	*•* Operationalise of models of care *•* Plan for succession *•* Provide professional and operational accountability and clinical governance.	*•* Develop their workforce *•* Develop a positive workplace culture *•* Meeting operational healthcare delivery targets *•* Invest in their workforce and infrastructure
C. Opinion Leaders *Come from wide range of roles and backgrounds *Individuals with informal influence on the attitudes and behaviours of others.*	Expert opinion leaders e.g., professional, and governing bodies *•* Professional growth advocacy *•* Protect professions’ credibility and standards *•* Strengthen professional collaborations and associations *•* Ensure public’s right to quality healthcare Peer opinion leaders (e.g., doctors, executives, researchers, executives, patient advocacy group members) *•* Ensure high standards of care *•* Ensure consumer needs are met *•* Advocate for healthcare professional needs	*•* Define scope of practice for professions *•* Shape understanding of gaps, opportunities, enablers, and barriers to implementation of professional role substitution *•* Garner respect and influence *•* Engage networks and interpersonal skills *•* Gain respect, trust and communicate experience and expertise, *•* Connect needs of healthcare, policy makers healthcare professionals and delivery services	*•* Exert influence through status, representation, and authority in their communities *•* Provide governance including accreditation, certification, registration, licencing *•* Provide continuing education and training for healthcare professionals. *•* Shape communication and implementation strategies	*•* Change laws and policies *•* Grow professions’ influence *•* Improve health care standards and ensure equitable access to quality, cost effective services *•* Deliver safe and quality healthcare
D. Implementation Facilitators *Individuals with subject matter expertise who assist, coach, or support implementation, e.g., clinical, and medical directors*	*•* Workforce shortage *•* Activity and performance demands *•* Deliver patient-centred and quality healthcare	*•* Provide training and upskilling opportunities. *•* Coach and support implementation (subject matter experts)	*•* Provide clinical governance safety oversight in models of care *•* Provide endorsement and support *•* Influence funding and resource allocation *•* Influence continuation/sustainability of models of care	*•* Improve patient experience *•* Support inter and transdiscipli*•* nary care *•* Increase skilled *•* workforce *•* Meet departmental goals and key performance indicators *•* Sustain and scale up of professional role substitution models
E. Implementation Leads/Delivers *Individuals who lead efforts to implement the innovation or are directly or indirectly delivering the innovation*	*•* Career advancement and personal development *•* Work satisfaction Deliver patient-centred and quality healthcare	*•* Deliver to full or extended scope *•* Collegiate with other healthcare professionals *•* Execute high-level communication and management skills	*•* Undertake extra training to extend scope of practice as required. *•* Seek funding to pilot and implement models of care *•* Champion legislation change if required	*•* Diversify roles *•* Improve patient journey, experience, and outcomes *•* Increase inter- disciplinary care *•* Enhance work relationships across disciplines *•* Invest in continuous learning and development
F. Implementation Support and Team Members *Individuals who collaborate with and support the Implementation Leads*	*•* Successful project implementation and delivery	*•* Project manage *•* Provide health analytics/digital, information technology and transformation *•* Administrate and allocate resources	*•* Manage funding to support implementation. *•* Build relationships in inner setting e.g., arranging meetings etc.	*•* Implement and deliver projects *•* Improve adoption of models of care *•* Improve sustainability and scale up of professional role substitution
G. Innovation Recipients *Individuals who are directly or indirectly receiving the innovation.*	*•* Timely access to care *•* Reassurance *•* Improved symptoms and quality of life *•* Affordable healthcare and healthcare related costs *•* Continuity of care *•* Improved understanding of healthcare conditions	*•* Communicate their experience of care *•* Share information on patient healthcare needs *•* Identify gaps in healthcare, helping ensure models of care are fit for purpose *•* Engage with recipients in the healthcare system	*•* Contribute to implementation and trial of new models of care *•* Influence perception and acceptability of professional role substitution models of care *•* Contribute to model of care evaluation	*•* Improve their patient experience and satisfaction *•* Experience holistic care *•* Obtain information on alternative healthcare management or treatments *•* Receive timely access to care *•* Receive reassurance *•* Reduce healthcare utilisation *•* Reduce healthcare related costs

## Implementation process

### Essential phases and strategies for effective implementation of allied professional role substitution healthcare models

Participants in our study provided insights into key stages necessary for implementing allied health professional role substitution models of care. We analysed their responses and mapped them to constructs in the implementation process domain of the CFIR, finding alignment with five out of the nine constructs. In the *planning* phase, participants emphasised the importance of conducting needs assessments and developing comprehensive implementation plans to identify gaps, set objectives, and consider resources and stakeholders' roles. One participant described, *"At the start-up of our model of care, we had a series of meetings involving all stakeholders... to develop very clear guidance and pathways for how patients would move through these services*" (P13, Allied Health Clinician).

*Engaging* was highlighted as crucial focused on involving diverse stakeholders, from healthcare providers to patients, forming multi-stakeholder teams to ensure a variety of perspectives and support for long-term sustainability. *" There were a broad range of stakeholders involved in the task force across Queensland Health and external to Queensland*." (P34, Workforce and Education) In the *doing* phase models of care often started as pilot projects, with services developing iteratively.

*Reflecting and evaluation* Participants stressed the importance of building evaluation into the model of care to ensure sustainability and strategic outcomes. However, challenges such as limited time and funding were acknowledged, as one participant stated, *"We don't get the time or the funding in my experienc*e" (P1, Allied Health Clinician). Lastly*,* in *adapting*, participants recognised the need for continuous learning and tailored strategies to the local context, acknowledging the necessity for flexibility in response to evolving healthcare systems. Moreover, strategies to enhance evaluation included dedicated funding, external evaluation to reduce bias, development of performance frameworks, and tailored technology and digital systems allowing data collection and analysis at the point of care. Collaboration with universities and the use of research frameworks and grants were also seen by participants as facilitators to enhance performance measurement.

## Implementation and innovation outcomes

### The assessment of outcomes derived from allied health professional role substitution models

**Fig. 2:**
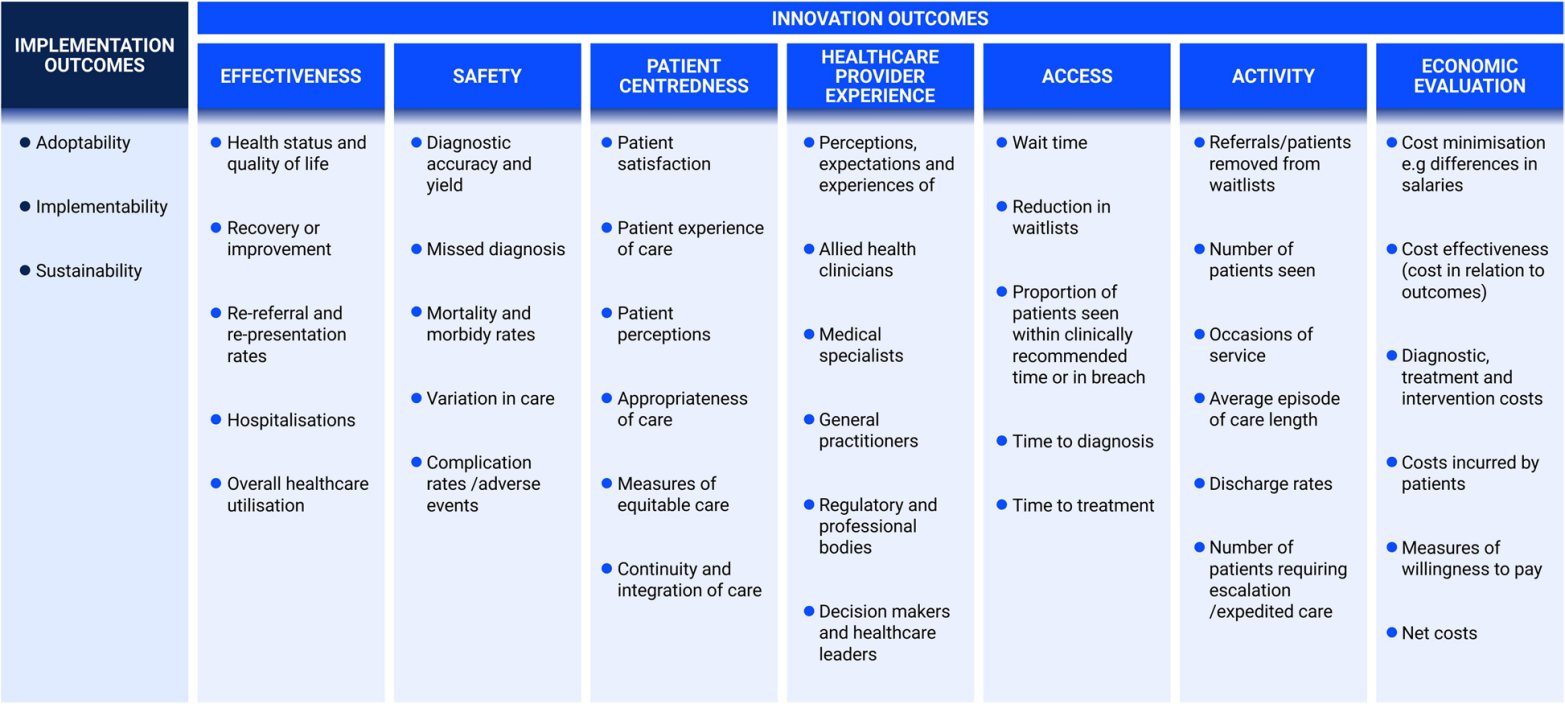
Recommended outcomes and examples to measure the impact of professional role substitution models of care

In our study, participants highlighted the importance of evaluating healthcare models' success and failure, focusing on both implementation process and innovation outcomes. They identified eight key domains, including implementation aspects such as adoptability, implementability, and sustainability, as well as innovation delivery outcomes like effectiveness, safety, patient-centeredness, healthcare provider experience, access, activity, and economic evaluation. One participant stressed the need for thoughtful measurement, stating*, “You do need to think about what you need to measure to prove the value of your service*.” (P35, Allied Health Leader). Figure 2 summarises these outcomes and provides examples of measures discussed by participants.

#### Implementation outcomes

Participants shared diverse perspectives on implementation success for allied health professional role substitution models, with factors like regulatory environment, financing, medical acceptance, stakeholder engagement, and individual characteristics playing key roles. Sustainability was particularly highlighted, as expressed by a participant, “*You need to know that a service that has been implemented is still running after several years*” (Participant 1, Allied Health Clinician).

#### Innovation outcomes

Participants emphasised specific outcomes in evaluating the impact of allied health professional role substitution models of care. One participant stressed the importance of measuring performance and demonstrating improved access and cost saving, saying, "*I think it is important to measure performance and to show that there is improved access and economic benefits. You know, to show that the service is doing what it was intended to do"* (P5, Allied Health Clinician). They also highlighted the need to track activity data, with another participant mentioning, *"We basically keep data on all of the occasions of service, how many patients are seen within the service, and how many patients are discharged."* (Participant 8, Allied Health Clinician).

Healthcare provider experience, including clinician and patient satisfaction, emerged as essential, with one participant suggesting*: "Surveying the general practitioners would be a good way of doing it as well, asking if they are happy with the service"* (P36, Executive Leader). Continuity of care and patient centredness were also emphasised. *"It's actually quite heartening hearing what our patients value and to see if our services line up with that*" (P29, Allied Health Clinician). Participants in the study also stressed the importance of safety as a crucial outcome measure in evaluating allied health professional role substitution models of care. One general practitioner (P7) highlighted this by stating, *"We need to know we are providing great healthcare to patients. You know that we are reducing harm, not causing harm, and hopefully not missing diagnoses”.*

Finally, participants perceived effectiveness as paramount for assessing the success and impact of the model on patient care experiences and health outcomes. One executive leader (P36) expressed: *"Forgetting about everything else, the patients' view of whether or not they've been treated adequately to me is the most important. If there are no outcomes with the model of care, the patients won't be satisfied, and they will say so."*

## Discussion

This study investigated factors influencing the implementation and performance evaluation of allied health professional role substitution models of care using the CFIR framework. We identified six overarching themes aligned with CFIR domains and outcomes. These themes covered dynamics such as innovation catalysts, evidence, advantages, and disadvantages; external factors affecting implementation and evaluation; internal structural, political, and cultural contexts; roles and contributions of individuals; essential implementation phases and strategies; and assessment of model outcomes. Our analysis identified twenty-seven underlying constructs and subconstructs within the CFIR framework that influence professional role substitution implementation. Additionally, we identified ten key constructs across implementation and innovation outcome categories: adoptability, sustainability, implementability, effectiveness, safety, patient-centeredness, accessibility, healthcare provider experiences, service delivery metrics, and economic evaluations. These findings addressed critical questions regarding factors influencing implementation and methods for assessing the impact of care models. Overall, this study provides a robust framework for implementing and evaluating allied health professional role substitution models, effectively addressing gaps in literature and practice.

### Priority areas of focus

While prior studies have demonstrated the potential benefits of these models in terms of providing safe, effective, and cost-efficient care, [10, 11] the current research goes further by exploring stakeholders' perceptions and experiences in depth. Grounded in the Consolidated Framework for Implementation Research (CFIR), [36–38] it explores the multifaceted factors influencing the adoption and integration of these models within healthcare systems.

Healthcare organisations play a significant role in either facilitating or impeding the implementation of professional role substitution models of care [41]. Along with previous research , this work underscores the significance of supportive organisational cultures, adequate resources, leadership commitment, and medical endorsement as critical factors for the successful adoption of such models [42]. Conversely, factors like resistance to change, resource limitations, and insufficient infrastructure can pose significant barriers that must be addressed to ensure successful implementation.

Traditional healthcare structures were once considered conducive to advancing medical sciences [43]. However, recent reviews have highlighted how entrenched organisational cultures and long-held traditions within healthcare settings may now act as barriers to alternative models of practice and hinder improvements in healthcare access for the community [41]. Consequently, healthcare organisations must proactively assess their readiness for new models and develop strategies to overcome these barriers. Leveraging the constructs and principles identified in the inner setting domain of this study is essential for cultivating a culture that fosters role substitution and innovation in healthcare delivery.

Stakeholders' perceptions and attitudes play a significant role in shaping the success of professional role substitution models of care, influenced by factors like medical buy-in, leadership support, and engagement strategies [41, 44, 45]. Effective stakeholder engagement strategies, alongside tailored training, communication programs and ongoing support mechanisms, emerge as crucial tools for addressing individual concerns and fostering buy-in from all involved parties. These findings align with similar studies in physiotherapy and nursing, emphasising the universal importance of considering individual perspectives in healthcare implementation efforts [41, 44–46].

This research emphasises the importance of incorporating perspectives from patients and innovation recipients to enhance the success of healthcare interactions. Integrating these viewpoints strengthens the potential for sustainable adoption of evidence-based innovations, promoting patient-centred care [47–49]. Patient involvement in co-designing and evaluating alternative healthcare models improves trust and acceptance, highlighting the significance of collaboration and patient engagement strategies for optimising implementation and evaluation processes [27, 28, 47].

Performance evaluation plays a pivotal role in assessing the implementation of professional role substitution models of care [25]. Monitoring various factors, including outcomes, patient satisfaction, quality of care, safety, healthcare professionals’ performance, healthcare system efficiency, and cost-effectiveness, can provide valuable insights for ongoing improvement, optimisation, and sustainability of models of care [25, 50]. We address gaps highlighted in previous research, particularly concerning the lack of comprehensive evaluations and guidance on outcome measures [10, 22, 25]. Many current frameworks lack specificity in identifying key metrics relevant to professional role substitution models [25, 26]. However, this study delineating eight key outcome measures emphasises a data-driven approach to decision-making. This represents an advancement in the field, providing a structured framework for assessing the impact and value of these models.

#### Implications for policy, practice and future research

In combination with existing literature in various alternative healthcare delivery models, this study highlights the shared challenges and opportunities across healthcare professions and settings [41, 45, 46]. Our analysis of implementation considerations, stakeholder perspectives, and outcome measures, advances theoretical understanding and also provides practical guidance for real-world implementation and evaluation. These insights can be extended beyond Australia's healthcare system, with implications for policy development, collaboration, knowledge exchange, and healthcare delivery practices in other regions.

In practice, maximising the effectiveness and sustainability of professional role substitution models necessitates comprehensive training and education initiatives for health professionals. Collaborating with professional bodies and universities can standardise training, provide continuous professional development opportunities, and address individual factors that impact implementation readiness for alternative healthcare delivery models.

Adapting regulatory frameworks to the evolving healthcare landscape is paramount, necessitating clear guidelines and legal frameworks to delineate practice boundaries and facilitate the seamless implementation of expanded roles. Adequate funding is critical to support various aspects, including staffing, infrastructure development, establishment of incentivising reimbursement models, research, evaluation, and ensuring ongoing sustainability. Prioritising evidence-based policy development, informed by comprehensive evaluation of care models, is essential to ensure alignment with best practices and standards of care. Integrating standard outcome measures into evaluation frameworks is crucial for accurately assessing the impact and effectiveness of care models, thereby enabling informed decision-making based on evidence. The research we have conducted supports these assertions, emphasising the importance of these factors for the successful implementation and sustainability of alternative healthcare delivery models.

Our findings may therefore serve as a catalyst for discussion and debate on allied health professional role substitution and other alternative healthcare delivery models, guiding future research endeavours. Exploring longitudinal studies to gauge sustainability and long-term impact, conducting comparative analyses across diverse settings and patient populations, and conducting qualitative inquiries to identify implementation and evaluation facilitators and barriers are critical. Additionally, research in health economics, health information technology, policy analysis, and interprofessional collaboration can provide valuable insights to optimise implementation practices and enhance the applicability of these models across different healthcare systems and cultural contexts.

### Strengths and limitations

The strengths of this study lie in the diverse range of stakeholders involved, including key opinion leaders, decision makers, allied health clinicians, medical professionals, policymakers, healthcare administrators, university partners, professional bodies, advocates, and patients. The inclusion of participants with varied experiences enhances the robustness of the findings. Purposeful sampling with maximum variation further improves the transferability of the results.

The use of the COREQ-checklist and independent co-coding and discussions among the research team enhance the credibility, trustworthiness, and transparency of the study [31]. Another notable strength is the use of the Consolidated Framework for Implementation Research (CFIR) to guide the analysis, which helped identify and organise themes into multi-level intervention principles that influence implementation effectiveness. It should be noted that the CFIR was not used to guide data collection, as is often practiced, [38] as this may have limited the exploration of qualitative themes relevant to the research question but not explicitly aligned with CFIR domains and constructs.

As the study was conducted in Australia, the generalisability of the findings to other stakeholders or healthcare contexts in different countries and settings may be limited. Additionally, as with any research involving human subjects, the possibility of self-selection bias influencing the results cannot be excluded, and the findings should be interpreted with this in mind. Insights gained from this study may also have broader implications for other countries facing similar challenges in healthcare delivery. By examining similarities and differences in healthcare systems and regulatory environments, countries can however learn from Queensland’s experiences adopting strategies to support the implementation of role substitution models.

## Conclusions

In conclusion, this study provides a systematic examination of the key elements and principles influencing the implementation and performance evaluation of professional role substitution models of care. By understanding the multifaceted nature of these factors and addressing the challenges and opportunities associated with expanded healthcare roles, healthcare systems can navigate complexities and capitalise on opportunities. This holistic approach, involving collaboration among stakeholders and considering patient safety, quality of care, and optimal healthcare outcomes will contribute to the development of more efficient, equitable, sustainable, and patient-centred models of care and healthcare systems.

### Supplementary Information

Supplementary Material 1.

Supplementary Material 2.

## Data Availability

Data is available from corresponding author on reasonable request.

## Abbreviations

CFIR: Consolidated Framework for Implementation Research
COREQ: Consolidated Criteria for Reporting Qualitative Research
Pas: Physician Assistants
