# Supplementary material for: Unlocking potential: a qualitative exploration guiding the implementation and evaluation of professional role substitution models in healthcare
Source: Implement Sci Commun. 2024 Jul 12;5:73. doi: 10.1186/s43058-024-00611-x (PMC11245812; doi:10.1186/s43058-024-00611-x)
Supplement: Supplementary file 2 — Supplementary Material 2. [file 43058_2024_611_MOESM2_ESM.docx]

|  |
| --- |

**Supplementary file 1: Semi-structured interview guide:** **Role Substitution of Specialist Medical Doctors with Allied-health Professionals – A Qualitative Exploration of Stakeholder Experiences and Perceptions of Performance Measurement**

**Introductory script**

- Thank participant for being involved in research
- Brief overview of the aims of the research project, their involvement, and how their privacy and confidentiality will be maintained
- Ask participant to raise any questions before commencing interview

**Interview questions**

1. Overview of stakeholder’s role within Queensland Health/ Health/HHS
2. To help us put our discussion into perspective, can you please give me an overview of your role?
3. Can you please describe what role/exposure *if any* that you have had with professional role substitution models of care?

*Prompts: inception/establishment, implementation, funding, evaluation*

1. Overview of perceptions, expectations, and experiences, of professional role substitution models of care
2. In your opinion, what role do professional role substitution models of care play in delivering or addressing key strategic areas in healthcare delivery- Local, State, National, Internationally

*Prompts: GCH Strategic Plan: Delivering world class care, Make the best use of our resources, drive future-focussed change. Show the participant strategic plan 2020-2024*

*Prompts: Department of health strategic plan 2021-2025 Promote and protect the health of all Queenslanders, Interconnected systems which drive co-designed models of care and care support pathways to support HHS, Support and advance workforce, Advance health equity for first nations people, Health reforms that plans for sustainable future*

*Prompts: AHHA 2016-2020: Equitable, Accessible, Equitable, Sustainable, Outcome focused*

1. What has been your experiences with professional role substitution models of care?

*Prompts: What are some of the positive aspects. What are some of the areas that need to be improved in the delivery of these models of care? What are some of the gaps? What are the opportunities? Are there any barriers or enablers? Is this a strategy you would support for healthcare service delivery. (What, how, why, when)*

1. Overview of perception and experience with performance measurement in healthcare and professional role substitution

An essential step in designing and implementation of value-based healthcare is measuring performance which include meaningful health outcomes of its care for patients and the cost of its services, learning from that information to drive ongoing improvements in care and efficiency.

1. What are your thoughts on current healthcare performance is measured?

*Prompts: How important or necessary is it? Why? Are there any benefits to measuring performance? Are there any negative aspects to measuring performance?*

1. What are your thoughts or experiences on how performance is measured in professional role substitution models of care?

*Prompts: Adequacy, methods, guidelines, frameworks, value, culture, resources (financial, human, digital), training, reporting, resulting actions?*

*Prompts: What are some of the enablers and barriers to healthcare performance? What can be done in your sphere of influence to help improve performance measurement and specifically in professional role substitution (strategies e.g. (frameworks, education and training, tool kit and resource development, digital platforms, financial incentives, financial resources). What can you advocate for?*

1. For professional role substitution to be a legitimate and compelling alternative for healthcare delivery, what outcomes would you like to see?

*Prompt: To demonstrate value what matters? What needs to be measured?*

*Prompt: Why is this important to you? Which of these are priority areas for you?*

*Prompt: How important are these aspects; effectiveness, safety, appropriateness and responsiveness, continuity and integration of care, access sand equity, cost efficiency and productivity/sustainability,*

1. Would you like to add any other thoughts of performance measurement in healthcare and professional role substitution models of care?

**Closing script**

Thank participant for the interview and ask if there have any further questions
